# Application of machine learning methods for predicting under-five mortality: analysis of Nigerian demographic health survey 2018 dataset

**DOI:** 10.1186/s12911-024-02476-5

**Published:** 2024-03-25

**Authors:** Oduse Samuel, Temesgen Zewotir, Delia North

**Affiliations:** School of Mathematics, Statistics and Computer Science, University of KwaZulu-Natal, 4001 Durban, South Africa

**Keywords:** Under-five mortality, Machine learning, Nigeria, Demographic and health surveys, Decision-making tools

## Abstract

**Background:**

Under-five mortality remains a significant public health issue in developing countries. This study aimed to assess the effectiveness of various machine learning algorithms in predicting under-five mortality in Nigeria and identify the most relevant predictors.

**Methods:**

The study used nationally representative data from the 2018 Nigeria Demographic and Health Survey. The study evaluated the performance of the machine learning models such as the artificial neural network, k-nearest neighbourhood, Support Vector Machine, Naïve Bayes, Random Forest, and Logistic Regression using the true positive rate, false positive rate, accuracy, precision, F-measure, Matthew’s correlation coefficient, and the Area Under the Receiver Operating Characteristics.

**Results:**

The study found that machine learning models can accurately predict under-five mortality, with the Random Forest and Artificial Neural Network algorithms emerging as the best models, both achieving an accuracy of 89.47% and an AUROC of 96%. The results show that under-five mortality rates vary significantly across different characteristics, with wealth index, maternal education, antenatal visits, place of delivery, employment status of the woman, number of children ever born, and region found to be the top determinants of under-five mortality in Nigeria.

**Conclusions:**

The findings suggest that machine learning models can be useful in predicting U5M in Nigeria with high accuracy. The study emphasizes the importance of addressing social, economic, and demographic disparities among the population in Nigeria. The study’s findings can inform policymakers and health workers about developing targeted interventions to reduce under-five mortality in Nigeria.

## Introduction

Under-five mortality (U5M) is a significant indicator for tracking children’s health and a measure of a country’s health development. U5M is the likelihood of children dying before reaching the age of 5 years [1]. The rates of U5M remain a pressing global concern, particularly in Sub-Saharan Africa, where millions of children face the awful reality of dying before celebrating their fifth birthday [2]. Despite tremendous progress in decreasing U5M rates internationally in recent years, Sub-Saharan Africa continues to carry the highest burden of child mortality [3].

Healthcare has advanced quickly in recent years because of the increased accessibility of large datasets and the development of robust computational approaches. Machine learning (ML) approaches have emerged as a promising tool for evaluating big datasets and detecting patterns that would otherwise go undetected [4]. Researchers and health workers can get meaningful insights into the determinants influencing U5M and develop tailored remedies to reduce it by taking advantage of these strategies. ML algorithms can learn from big datasets automatically, enabling them to detect trends, correlations, and risk factors that humans might overlook [5]. By understanding the complex interactions among these risk factors, ML models can accurately predict which children are at a higher risk of dying before their fifth birthday [6, 7].

The ML method offer new opportunities for improving existing methods for predicting U5M. Statistical models have traditionally been used to examine data and identify risk factors [8–11]. This approach, however, frequently make oversimplified assumptions or fails to reflect complicated connections [12]. On the other hand, ML can handle non-linear correlations, account for interactions between multiple components, and adapt to changing patterns in data. Researchers can improve the accuracy and validity of their predictions by combining ML techniques into existing procedures, leading to more effective preventative and intervention measures to lower U5M rates [13]. Application of ML algorithms for predicting U5M can potentially enhance healthcare resource allocation. Health experts may more efficiently distribute resources such as medical staff, vaccinations, and other treatments by identifying high-risk areas or groups [14, 15]. Furthermore, ML may help with focused intervention programs by detecting certain risk factors prominent in specific population.

The purpose of this present study is to develop an efficient risk prediction model for U5M based on a more comprehensive dataset that includes an individual’s demographic and socioeconomic characteristics. Therefore, this study aims to identify the primary determinants of U5M and using the most relevant determinants, we seek to find the most effective ML model for U5M risk prediction.

## Methods

### Study setting

Nigeria is a West African nation with a total size of 923,768 square kilometres, ranking it 14th in Africa by landmass. It is bounded to the north by Niger, to the west by Benin, to the east by Cameroon, and the south by the Atlantic Ocean. Nigeria is Africa’s most populated nation, with an estimated 218 million inhabitants in 2022 [16]. In addition, the nation is home to about 250 ethnic groups and over 500 languages.

Nigeria is situated between the latitudes of 4°16’ and 13°53’ north and the longitudes of 2°40’ and 14°41’ east. Abuja, the country’s capital, is situated in the country’s centre. Nigeria has 36 states and the Federal Capital Territory. The states are subdivided further into local government areas.

Nigeria is a multifaceted nation with a long history and culture. The economy of the country is primarily on oil and gas, although agriculture is also significant. Nigeria is a United Nations, African Union, and Commonwealth of Nations member.

### Data source

In developing countries like Nigeria, where there is not enough reliable and sufficient vital registration system, the Demographic and Health Surveys become the major sources for acquiring information on U5M estimates. The data used in the present study was obtained from the most recent and available Nigeria Demographic and Health Surveys (NDHS) 2018 [17]. The NDHS is representative across the country with respondents drawn from all eligible women of childbearing age (15–49 years) living in the selected households. The survey presents current information on demographic and socioeconomic characteristics, and other health indicators in Nigeria, such as child and maternal mortality. Respondents supplied information on all children they gave birth to during the past five years prior to the survey. The full report on the methods and techniques used to gather data for the 2018 NDHS is available elsewhere [17].

### Sampling

The 2018 NDHS sample was a stratified sample chosen in two stages. Each of the 36 states and the Federal Capital Territory was stratified by dividing them into urban and rural areas. There were 74 sampling strata in total. A two-stage selection was used to select samples independently in each stratum. At each of the lower administrative levels, implicit stratifications were achieved by sorting the sampling frame before sample selection according to administrative order and using a probability proportional to size selection during the first sampling stage.

In each of the selected enumeration areas, a household listing operation was carried out, and the resulting lists of households served as a sampling frame for the selection of households in the second stage. In the second stage, a fixed number of 30 households were chosen from each cluster using equal probability systematic sampling, yielding a total sample size of approximately 42,000 households [17].

### Data

For this study, the sample used was 29, 992 women of reproductive age who had given birth in the past 5 years before the survey. However, to limit a recall bias, the most recent delivery the women had in the past 5 years before the study was considered.

### Ethical consideration

Ethical approval for this study was not necessary as the data used was secondary. The NDHS which is the source of the data had ethical approval before carrying out the survey. The data used in this study contains no identifiable information from the respondents.

### Conceptual framework

**Fig. 1:**
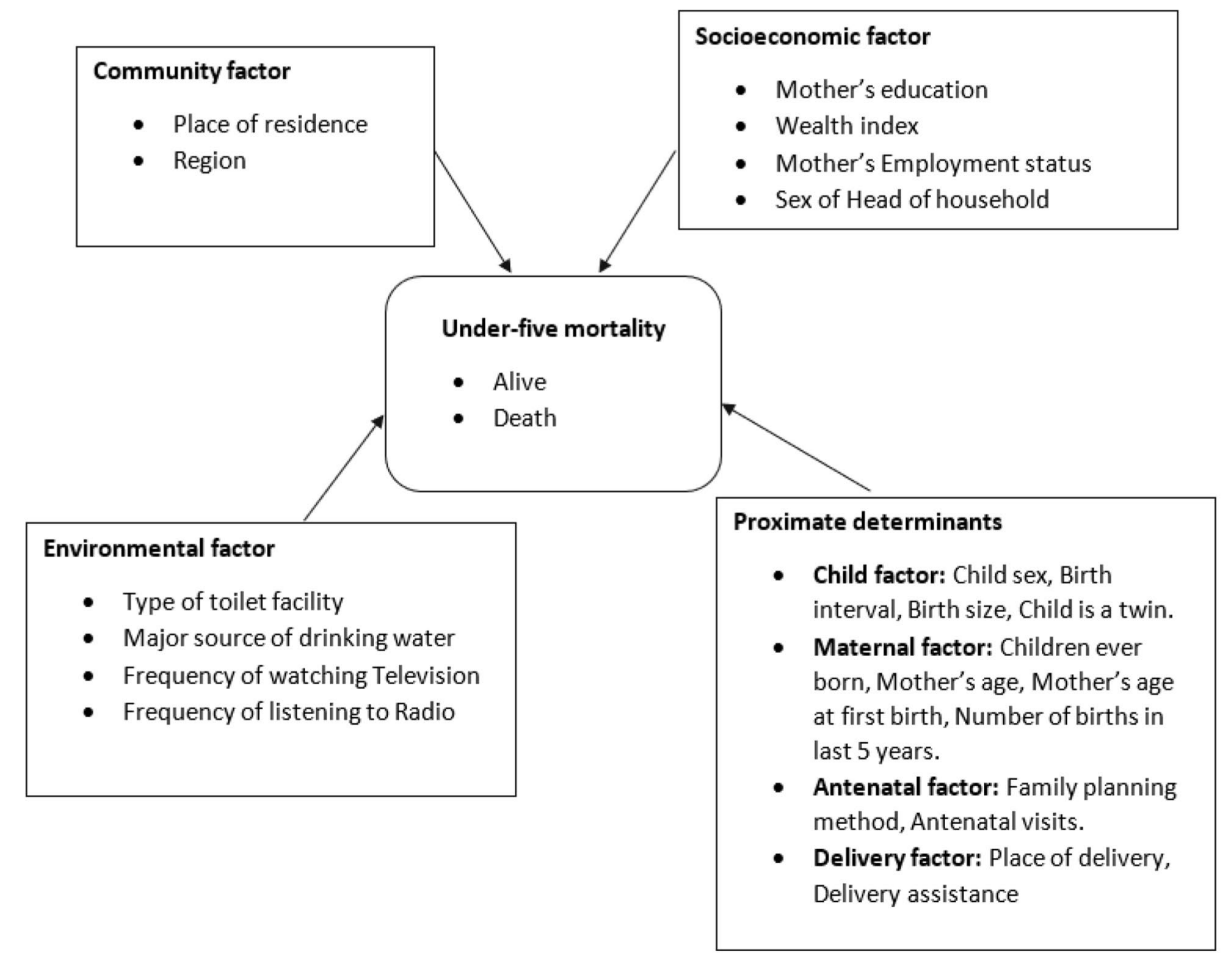
Conceptual framework for under-five mortality

Based on the information available in the NDHS 2018 datasets, the conceptual framework devised by Mosley and Chen for the study of child survival in developing countries [18] was modified and adopted in this study. Figure 1 shows the framework used in this study with potential indicators of U5M in Nigeria that were selected for investigation.

### Study variables

The outcome variable for this study was U5M, defined as the probability of a child born alive to reach the age of 5. The outcome was binary and labelled as “No” if the child is alive after their fifth birthday or “Yes” otherwise.

The independent variables included birth interval, child sex, birth size, child is a twin, delivery assistance, place of residence, family planning method, region, children ever born, birth order, antenatal visits, mother’s education, wealth index, mother’s age at first birth, type of toilet facility, mother’s age, mother’s employment status, number of births in last 5 years, major source of drinking water frequency of watching television and frequency of listening to radio. The selection of those variables was based on the conceptual framework and literature review of U5M [19–22].

### Data pre‑processing

Data pre-processing is a necessary technique used in transforming raw data into a meaningful and understandable structure. It is a necessary step in eradicating unnecessary, duplicated, and unreliable information from the data, and it is helpful in resolving discrepancies in the dataset. In this paper, data pre-processing was performed before training the ML models. First, records with more than 50% missing values such as postnatal check, health card and vaccination were excluded from the dataset. The remaining missing values in the dataset were handled using the imputation method. The Gini information method was used to identify the most important factors that contribute to U5M.

One of the key challenges of the ML method is the imbalanced data problem. This occurs when there are not an equal number of samples in each class. In the selected dataset, the outcome classes are significantly imbalanced, with 27,924 samples in the “No” class and only 2068 samples in the “Yes” class. This means that the trained algorithms are more likely to be biased towards the majority class (“No”), and the ML algorithms are more likely to categorize new observations as alive.

The problem of imbalanced data in this study was addressed using the SMOTE (Synthetic Minority Oversampling Technique) method. The SMOTE algorithm is an oversampling approach that generates synthetic samples for the minority class by selecting instances of the minority class that are close in the feature space, drawing a line between the instances in the feature space and drawing a new instance at a point along that line [23]. To create a synthetic sample, a random data instance from the minority class is first chosen, and then k of the nearest neighbours for that instance is found. A randomly selected neighbour is chosen, and a synthetic instance is created at a randomly selected point between the two instances in feature space [24]. This process is repeated until the minority class is balanced with the majority class.

### Model building

Univariate logistic regression was carried out to identify factors that are independently associated with U5M, and then to account for the relationship between different independent variables, we also performed a multivariate logistic regression. By performing univariate and multivariate logistic regression as baseline models, we can evaluate the performance of more complex ML algorithms. And we can gain insights into the data, select relevant variables, interpret results, and address data limitations.

The purpose of developing the predictive classifier models was to accurately predict U5M. Firstly, we randomly split the dataset into two sets: a training set and a test set. The training set was used to train the model, and the test set was used to evaluate the model’s performance. We used a 70/30 split, meaning that 70% of the data was used for training and 30% was used for testing. We repeated this process with an 80/20 split and then 10-fold cross validation to assess the impact of different training and testing ratios on the performance of the ML models.

We reviewed related studies on mortality [12, 25–27] to select suitable machine ML algorithms and considered the type and quality of the selected dataset during the modelling stage. To construct the mortality prediction model, we utilized seven ML algorithms. The algorithms were chosen after careful consideration of several key factors, including the nature of the problem, the characteristics of the dataset, and the goals of our research. We provide a detailed rationale for selecting the seven machine learning algorithms below:

#### J48 decision tree

For classification tasks, decision trees are both interpretable and effective. J48, a C4.5 algorithm implementation, was chosen for its ability to handle both numerical and categorical data [28]. Its decision rules shed light on the factors that influence U5M, making it useful for public health interpretation.

#### Artificial neural network (ANN)

ANNs have proven models for capturing complex data relationships [29]. Child mortality prediction may involve intricate patterns that ANNs can effectively learn and model. Because ANNs are non-linear, they can represent intricate dependencies in data, potentially capturing intricate factors contributing to U5M [30].

#### k-Nearest neighbour (k-NN)

k-NN is a flexible algorithm that can be used for both regression and classification tasks. In the prediction of child mortality, neighbouring instances may share similar characteristics, and k-NN can take advantage of this local information. It works especially well when the dataset contains local clusters of high and low mortality rates [31].

#### Support Vector Machine (SVM)

SVM is well-known for its ability to handle high-dimensional data and determine optimal hyperplane boundaries. SVM was chosen because of its ability to identify complex relationships in the dataset and create clear decision boundaries, potentially capturing non-linear interactions in the predictors [32].

#### Nave bayes (NB)

NB is a probabilistic algorithm that assumes predictor independence. While this assumption may not always hold in practice, NB is computationally efficient and especially useful when dealing with large datasets [33]. It serves as a baseline model, providing a simple yet effective approach to predicting U5M.

#### Random forest (RF)

The RF is an ensemble learning method that combines the predictive power of several decision trees [34]. Child mortality prediction frequently involves complex interactions, which RF can capture by aggregating the outcomes of various decision trees, improving overall model robustness and accuracy.

#### Logistic regression (LR)

The LR is a well-known binary classification algorithm. It is interpretable and provides information about how each predictor affects the outcome. The LR aids in determining the relative importance of various variables in influencing mortality rates in U5M prediction [35].

The combination of these seven algorithms had been designed to capitalize on the strengths of each method while improving the overall predictive performance of our child mortality model. This method ensures a thorough examination of the dataset’s characteristics and promotes a better understanding of the factors that influence under-five mortality.

### Performance evaluation

Model performance evaluation is essential for developing an effective ML model. We evaluated the performance of the ML models using a confusion matrix, which visualizes the actual and predicted class accuracies. The confusion matrix shows how many times the models predicted correctly and incorrectly. The predicted values are categorized as True positive (TP), False Positive (FP), True Negative (TN), and False Negative (FN). We then measured the model’s performance using True Positive Rate (TPR) also known as Sensitivity, False Positive Rate (FPR), accuracy, precision, F-measure, and the Matthews correlation coefficient (MCC).

Another evaluation metric used was the Area Under the Receiver Operating Characteristics (AUROC) which tells how much the models are capable of distinguishing between U5M classes. We compared the evaluation criteria to determine the best model for predicting U5M.

The descriptive and data pre-processing was performed using SPSS 14 software. The ML algorithms were achieved using Weka (v3.9.2) software.

## Result

### Descriptive results of the background characteristics

Table 1 presents the prevalence of U5M based on the sample characteristics. Of the 29, 992 children in the sample, around 6.9% died before their fifth birthday. The results show that U5M rates vary significantly across different characteristics. For example, the U5M rate is higher among children born to mothers aged 35–49 years (9.9%) compared to those born to mothers aged 20–34 years (5.4%) or 15–19 years (6.3%). Similarly, the U5M rate is higher in rural areas (7.6%) compared to urban areas (5.7%). The North-West region has the highest U5M rate (10.1%), while the South-West region has the lowest (3.9%).

Maternal education is also found to be a significant factor affecting U5M rates. Children born to mothers with no education have a higher under-five mortality rate (9.0%) compared to those born to mothers with primary education (7.3%) or secondary/higher education (4.6%). The employment status of the woman is another factor that affects U5M rates. Children born to unemployed women have a higher U5M rate (7.7%) compared to those born to employed or self-employed women (6.6%).

**Table 1:** Descriptive statistics of U5M outcome by background characteristics

Characteristics	Under-five mortality		*p*-value
	No (percent)	Yes (percent)	
**Maternal age**			< 0.001
15–19	93.7	6.3	
20–34	94.6	5.4	
35–49	91.5	8.5	
**Residence**			< 0.001
Urban	94.3	5.7	
Rural	92.4	7.6	
**Region**			< 0.001
North-Central	94.0	6.0	
North-East	92.0	8.0	
North-West	89.9	10.1	
South-East	94.8	5.2	
South-South	95.5	4.5	
South-West	96.1	3.9	
**Maternal education**			< 0.001
No education	91.o	9.0	
Primary	92.7	7.3	
Secondary/Higher	95.4	4.6	
**Employment status of woman**			0.002
Unemployed	92.3	7.7	
Employed/ self-employed	93.4	6.6	
**Wealth index**			< 0.001
Poor	91.2	8.8	
Middle	93.2	6.8	
Rich	95.3	4.7	
**Sex of child**			0.006
Male	92.7	7.3	
Female	93.5	6.5	
**Childbirth interval**			< 0.001
First birth	94.0	6.0	
Less 24 months	90.6	9.4	
24 months and over	93.5	6.5	
**Child is a twin**			< 0.001
Single	93.5	6.5	
twin or multiple birth	74.5	25.5	
**Birth size**			< 0.001
Large	93.6	6.4	
Average	93.1	6.9	
Small	91.6	8.4	
**Sex of Head of household**			0.037
Male	93.0	7.0	
Female	93.8	6.2	
**Age of woman at first birth**			< 0.001
Less than 20 years	91.8	8.2	
20–25 years	94.5	5.5	
26 + years	95.7	4.3	
**Children ever born**			< 0.001
1–3	94.7	5.3	
4–5	94.5	5.5	
Over 5	89.3	10.7	
**Number of births in last 5 years**			0.049
0–2	93.2	6.8	
Over 2	91.9	8.1	
**Family planning method**			< 0.001
No method	92.4	7.6	
Folkloric or traditional method	96.9	3.1	
Modern method	96.4	3.6	
**Place of delivery**			0.573
Home	93.0	7.0	
Health facility	93.2	6.8	
**Antenatal visits**			< 0.001
None	91.2	8.8	
1	93.8	6.2	
2–3	93.6	6.4	
4+	93.9	6.1	
**Skilled assistance during delivery**			< 0.001
No	92.3	7.7	
Yes	94.8	5.2	
**Type of toilet facility**			< 0.001
Improved	95.5	4.5	
Not improved	91.8	8.2	
**Major source of drinking water**			0.277
Improved	93.6	6.4	
Not improved	93.0	7.0	
**Frequency of watching Television**			< 0.001
Less than once a week	92.2	7.8	
Once a week or more	95.4	4.6	
**Frequency of listening to Radio**			0.029
Less than once a week	92.9	7.1	
Once a week or more	93.6	6.4	

### Univariate and multiple logistic regression analysis of factors associated with U5M

Table 2 shows both Univariate and multivariate logistic regression that reports the odds ratio and adjusted odds ratio along with their 95% CI and *p*-value of various variables associated with U5M. Each variable has a reference category, and the odds ratio or adjusted odds ratio is reported relative to that reference category. The *p*-value indicates whether the odds ratio or adjusted odds ratios are statistically significant.

For maternal age, the odds of U5M are higher for mothers aged 20–34 and 35–49 compared to mothers aged 15–24. The adjusted odds ratio was 2.00 (95% CI: 1.82–2.20) for mothers aged 20–34 and 3.76 (95% CI: 3.38–4.19) for mothers aged 35–49, with a *p*-value of < 0.001. For maternal education, the odds of U5M are higher for mothers with no education or primary education when compared to mothers with secondary or higher education. The adjusted odds ratio was 2.67 (95% CI: 2.39–2.99) for mothers with no education and 2.21 (95% CI: 1.95–2.51) for mothers with primary education, with a *p*-value of < 0.001.

For residence, the adjusted odds ratio was 1.28 (95% CI: 1.17–1.39) for children living in rural areas compared to urban areas, with a *p*-value of < 0.001 indicating U5M odds are higher in rural areas compared to urban areas. The odds of U5M vary significantly by region. Compared to the North-West region (reference category), the odds ratio for U5M is lower in the North-Central, North-East, South-East, South-South, and South-West regions. The *p*-value for all regions is < 0.001.

For employment status, the adjusted odds ratio was 3.91 (95% CI: 3.69–4.15) for unemployed mothers compared to employed or self-employed mothers, with a *p*-value of < 0.001 signifying a higher risk of U5M for unemployed women compared to employed or self-employed women. The odds of U5M are higher for poor and middle-class families compared to rich families, with an adjusted odds ratio of 1.60 (95% CI: 1.39–1.85) for children from poor households and 5.12 (95% CI: 4.51–4.81) for children from middle-class households when compared to those from rich households, all with a *p*-value of < 0.001.

For sex of child, the adjusted odds ratio was 2.28 (95% CI: 2.15–2.41) for female children compared to male children, with a *p*-value of < 0.001 implying that female children have higher odds of U5M when compared to male children in the sample. For the childbirth interval, first-birth children have increased odds of U5M with an adjusted odds ratio of 5.05 (95% CI: 4.57–5.58) and a *p*-value of < 0.001 when compared to those with an interval of 24 months and above. Similarly, children with a birth interval less than 24 months have increased odds (AOR = 3.79; 95% CI: 3.55–4.05) of U5M when compared to children with a birth interval above 24 months.

**Table 2:** Univariate and Multivariate logistic regression for factors associated with U5M

Variable	OR	95% CI	AOR		95% CI	
**Maternal age (ref: 15–24)**						
20–34	1.00**	(0.95–1.05)	2.00		(1.82–2.20)	
35–49	1.63**	(1.55–1.71)	3.76**		(3.38–4.19)	
**Residence (ref: Urban)**						
Rural	5.41**	(5.17–5.66)	1.28**		(1.17–1.39)	
**Region (ref: North-West)**						
North-Central	0.40**	(0.38–0.42)	0.89**		(0.82–0.97)	
North-East	0.64**	(0.62–0.67)	0.66**		(0.62–0.71)	
South-East	0.14**	(0.13–0.15)	0.87**		(0.75–1.01)	
South-South	0.11**	(0.1–0.12)	0.27**		(0.23–0.32)	
South-West	0.06**	(0.06–0.07)	0.88**		(0.74–1.05)	
**Maternal education (ref: Secondary/Higher)**						
No education	22.46**	(21.01-24)	2.67**		(2.39–2.99)	
Primary	6.79**	(6.29–7.34)	2.21**		(1.95–2.51)	
**Employment status of woman (ref: Employed/self-employed)**						
Unemployed	5.77**	(5.56–5.99)	3.91**		(3.69–4.15)	
**Wealth index (ref: Rich)**						
Poor	5.65**	(5.14–6.2)	1.60**		(1.39–1.85)	
Middle	34.67**	(31.93–37.65)	5.12**		(4.51–5.81)	
**Sex of child (ref: Male)**						
Female	2.1**	(2.03–2.17)	2.28**		(2.15–2.41)	
**Childbirth interval (ref: 24 months and above)**						
First birth	1.60**	(1.52–1.67)	5.05**		(4.57–5.58)	
Less 24 months	3.92**	(3.77–4.09)	3.79**		(3.55–4.05)	
**Child is a twin (ref: Single birth)**						
twin or multiple birth	4.96**	(4.5–5.46)	4.58**		(3.90–5.37)	
**Birth size (ref: Large)**						
Average	2.88**	(2.73–3.03)	5.48**		(5.06–5.93)	
Small	12.25**	(11.5-13.06)	14.3**		(13.01–15.71)	
**Sex of Head of household (ref: Male)**						
Female	0.29**	(0.27–0.31)	0.70**		(0.62–0.78)	
**Age of woman at first birth (Less than 20)**						
20–25 years	0.68**	(0.66–0.71)	1.63**		(1.52–1.75)	
26 + years	0.25**	(0.23–0.27)	1.43**		(1.24–1.65)	
**Children ever born (ref: 1–3)**						
4–5	1.80**	(1.71–1.89)	1.65**		(1.51–1.81)	
Over 5	6.80**	(6.52–7.1)	3.66**		(3.38–3.97)	
**Number of births in last 5 years (ref: 0–2)**						
Over 2	5.26**	(4.95–5.58)	3.37**		(3.08–3.69)	
**Family planning method (ref: Modern method)**						
No method	3.02**	(2.56–3.57)	4.03**		(3.04–5.35)	
Folkloric or traditional method	12.56**	(11.21–14.08)	3.61**		(3.04–4.29)	
**Place of delivery (ref: Health facility)**						
Home	9.38**	(8.99–9.79)	4.01**		(3.73–4.30)	
**Antenatal visits (ref: 4+)**						
0	10.05**	(9.63–10.49)	2.98**		(2.78–3.18)	
1	6.84**	(6.13–7.64)	2.55**		(2.17–2.99)	
2–3	3.25**	(3.05–3.46)	1.20**		(1.09–1.32)	
**Skilled assistance during delivery (ref: Yes)**						
No	10.41**	(9.78–11.07)	1.21**		(1.09–1.35)	
**Toilet type (ref: Improved)**						
Not improved	15.29**	(13.97–16.72)	1.54**		(1.32–1.80)	
**Major source of drinking water (ref: Improved)**						
Not improved	2.57**	(2.41–2.76)	1.36**		(1.21–1.53)	
**Frequency of watching television (ref: Once a week or more)**						
Less than once a week	12.86**	(11.94–13.86)	1.21**		(1.06–1.38)	
**Frequency of listening to Radio (ref: Once a week or more)**						
Less than once a week	5.17**	(4.91–5.45)	1.87**		(1.71–2.04)	

** The OR is significant at 0.01 level

### Developing and evaluating models

To develop a predictive model for U5M, seven ML models (NB, LR, SVM, k-NN, ANN, J48, and RF) were applied. The models were trained and validated using three different data splits: 70% training and 30% validation, 80% training and 20% validation, and 10-fold cross-validation. The performance of each model was evaluated using metrics discussed in previous section. The results from Table 3 showed that all three data splits produced nearly equal performance metrics for all seven models. However, after careful consideration, the70–30% split was chosen based on established research that favours this ratio.

From Table 3, the ANN, J48, and RF models recorded the lowest FP rates with a value of 0.11. The RF had the highest performance in terms of TP rate, precision, F-measure, and accuracy. It achieved a TP rate of 0.9, precision and F-measure of 0.9, and accuracy of 89.47%. The RF model also had the highest performance in terms of MCC and AUROC. It achieved an MCC of 0.79 and an AUROC of 0.96. Overall, the RF model followed by the ANN model performed well in all measures, indicating that they are effective in predicting U5M.

**Table 3:** Performance evaluation of the selected ML algorithms for U5M prediction

Training-Test ratio	Measures	NB	LR	SVM	k-NN	ANN	J48	RF
70/30	TP Rate	0.82	0.87	0.87	0.88	**0.89**	0.88	**0.89**
	FP Rate	0.18	0.13	0.13	0.12	**0.11**	0.12	**0.11**
	Precision	0.84	0.87	0.87	0.88	**0.89**	0.88	**0.89**
	F-Measure	0.82	0.87	0.87	0.88	**0.89**	0.88	**0.89**
	MCC	0.66	0.75	0.75	0.77	0.77	0.77	**0.79**
	Accuracy	82.18	87.32	87.3	88.26	88.71	88.35	**89.25**
	AUROC	0.92	0.95	0.87	0.94	**0.96**	0.93	**0.96**
80/20	TP Rate	0.82	0.87	0.87	0.88	0.89	0.89	**0.90**
	FP Rate	0.18	0.13	0.13	0.12	**0.11**	**0.11**	**0.11**
	Precision	0.84	0.87	0.88	0.88	0.89	0.89	**0.9**
	F-Measure	0.82	0.87	0.87	0.88	0.89	0.89	**0.9**
	MCC	0.66	0.75	0.75	0.77	0.78	0.77	**0.79**
	Accuracy	82.00	87.39	87.43	88.41	88.82	88.61	**89.47**
	AUROC	0.91	0.95	0.87	0.94	**0.96**	0.94	**0.96**
10-fold	Sensitivity	0.82	0.87	0.88	**0.89**	**0.89**	**0.89**	**0.89**
	FP Rate	0.18	0.13	0.13	0.12	**0.11**	**0.11**	**0.11**
	Precision	0.84	0.87	0.88	**0.89**	**0.89**	**0.89**	**0.89**
	F-Measure	0.82	0.87	0.88	**0.89**	**0.89**	**0.89**	**0.89**
	MCC	0.65	0.75	0.75	0.77	0.78	0.77	**0.79**
	Accuracy	81.95	87.29	87.47	88.49	89.02	88.58	**89.4**
	AUROC	0.91	0.95	0.88	0.94	**0.96**	0.94	**0.96**

### Variable importance

**Fig. 2:**
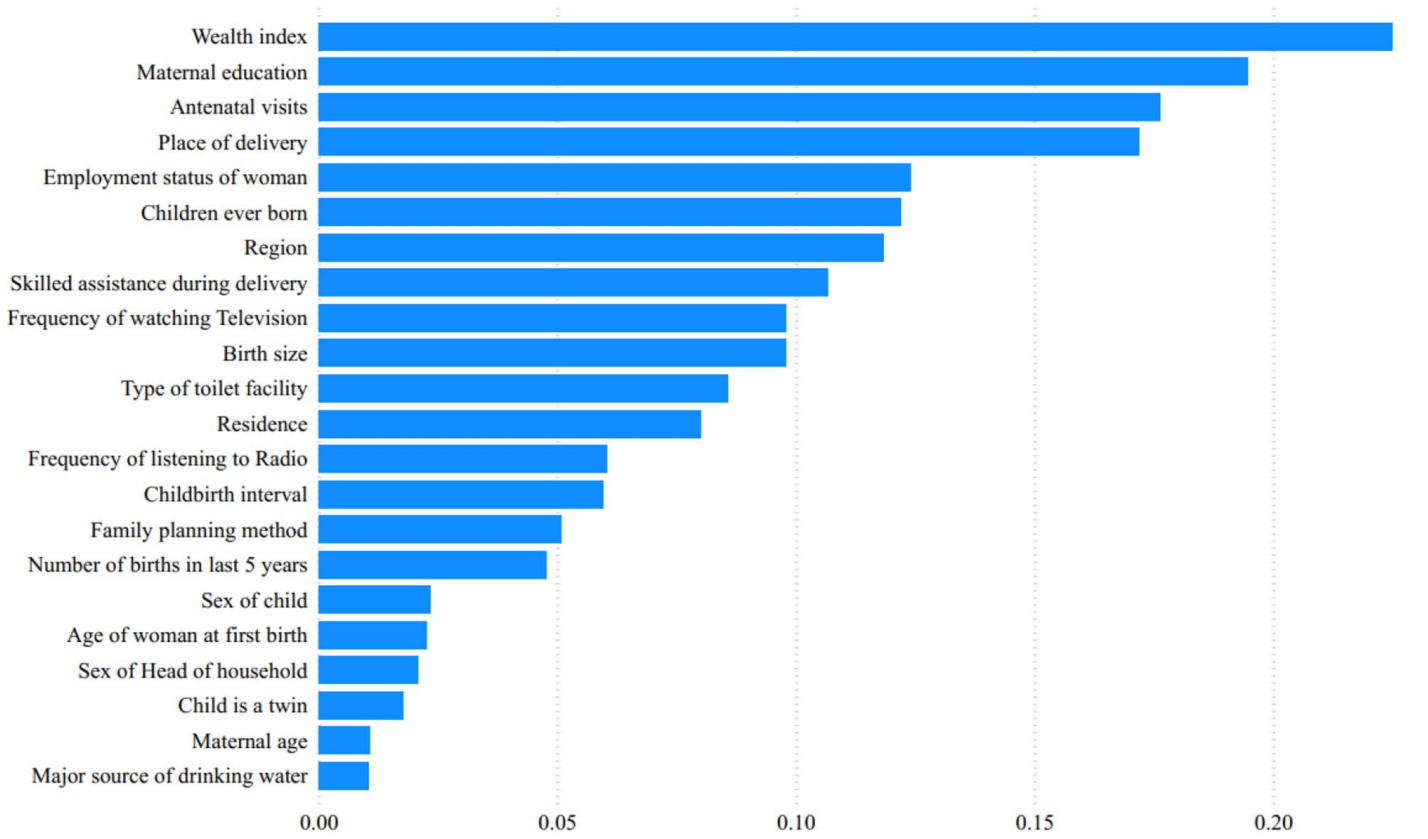
Variable importance measures from the random forest model

In ML prediction, it is important to identify the most important variables in the data. This can be done using a variety of methods, but in this study, we used the information gain rank method to identify the most important variables associated with U5M. The results, shown in Fig. 2, indicate that the top 10 variables that contribute the most to U5M are wealth index, maternal education, antenatal visits, place of delivery, employment status of the woman, children ever born, region, skilled assistance during delivery, frequency of watching television, and birth size.

## Discussion

This study demonstrates the implementation of ML techniques for predicting U5M rates in Nigeria. It is the first study to employ ML methods for predicting U5M rates in Nigeria with nationally representative data. The study showcases the superior predictive capabilities of ML methods as compared to other conventional approaches in identifying factors linked to U5M. This is not surprising, as ML methods have been shown to outperform traditional statistical methods in several fields of medicine [36]. The findings of this study are consistent with the findings of previous studies, which have shown that ML methods can be used to predict mortality with high accuracy [37–39].

The RF algorithm performed better than the other ML algorithms in predicting U5M. The RF algorithm achieved the highest performance in terms of MCC (0.79). The MCC is a measure of the quality of binary classifications, and it ranges from − 1 to 1, with 1 indicating perfect prediction. The high value of MCC for the RF algorithm indicates that it is effective in predicting U5M. Other studies have demonstrated the capability of RF in accurately predicting mortality rates [40–42]. Based on the AUROC measure, both the RF and ANN algorithms performed better than the other ML algorithms in predicting U5M. The high value of AUROC (0.96) for both RF and ANN indicates that they are effective in predicting U5M. The other ML algorithms, including NB, LR, SVM, k-NN, and J48, achieved similar performance in terms of TP rate, FP rate, precision, F-measure, and accuracy.

Overall, the RF algorithm performed better than the other machine learning algorithms in predicting U5M, based on the MCC measure. However, the other algorithms achieved similar performance in terms of other measures, indicating that they are also effective in predicting U5M. It is important to note that the performance of these models may vary depending on the dataset and the specific problem being addressed. Therefore, it is recommended to evaluate the performance of multiple models and select the one that performs the best for a given problem.

The information gain method was used to identify the most important variables for predicting U5M. The results showed that the top ten important predictors are: wealth index, maternal education, antenatal visits, place of delivery, employment status of the woman, number of children ever born, region, skilled assistance during delivery, frequency of watching television, and birth size. These findings are consistent with the findings of previous studies, which have shown that these factors are all important predictors of U5M [43–45]. The variable importance results support the findings of the conventional logistic regression analysis in this study, which found that wealth index, maternal education, antenatal visits, place of delivery, employment status of the woman, children ever born, region, skilled assistance during delivery, frequency of watching television, and birth size all have a significant impact on U5M in Nigeria.

According to the conventional logistic regression findings, the chance of U5M decreases with maternal education. women with no education had the highest likelihood of having U5M, whereas women with secondary/higher education have the lowest likelihood. This might imply that with increasing understanding of health and hygiene habits, better access to healthcare, and higher economic status, the chances of U5M will be greatly decreased. It was discovered that children born to unemployed women are more likely to experience U5M compared to children born to employed or self-employed women. This suggests that economic empowerment and access to resources for women and their families can enhance maternal and child health outcomes, which can help to reduce the risk of U5M.

The risk of U5M decrease with increasing wealth. Poor and middle-class families have higher odds of under-five mortality compared to rich families. This higher risk of U5M for middle- and low-income families could be related to limited access to healthcare, poor nutrition, and inadequate living conditions. Regional differences in health outcomes and access to healthcare services can also influence the risk of U5M. Findings show children born in the North-west region have the highest likelihood of experiencing U5M while children born in the South-West region have the lowest likelihood of experiencing U5M. This is not surprising as the North-West region of Nigeria is the region with the highest population and increased rate of poverty while the opposite can be said of the South-West region of Nigeria. Addressing regional disparities in healthcare services and improving access to healthcare services can assist in combating the high U5M rate in Nigeria.

Children born to women who have given birth to more than five children are more likely to experience under-five mortality compared to children born to women who have given birth to 1–3 children. This finding suggests that family planning and access to reproductive health services can help to prevent unintended pregnancies and improve maternal and child health outcomes, which in turn decreases the risk of U5M. Our findings indicated that mothers who did not have any antenatal visits during their pregnancy are more likely to experience U5M compared to mothers who had four or more antenatal visits. This implies that antenatal care is an important factor in reducing the risk of U5M. Antenatal care provides an opportunity to identify and manage potential complications during pregnancy and childbirth, which can help to prevent or reduce the risk of U5M.

The result from the study shows higher risk of U5M for children born at home when compared to children born at a health facility. Delivery at a health facility with skilled birth attendance and access to emergency obstetric care can help to prevent or manage potential complications during childbirth, which reduces the risk of U5M.

## Conclusion

The main goal of this study was to compare and assess the effectiveness of various machine learning (ML) algorithms in predicting under-five mortality in Nigeria while considering the impact of different train-test split ratios. Standard evaluation metrics were used to assess the predictive power of the ML models under various testing and training ratios. Our findings confirm that ML models can accurately predict the U5M, which suggests their potential usefulness in decision-making tools for relevant organizations. The analysis of different machine learning algorithms for under-five mortality estimation has revealed that the best-performing models are Random Forest (RF) and Artificial Neural Network (ANN). Their consistent outstanding performance across multiple evaluation metrics and training-test ratios leads to this conclusion.

The use of the best-performing models for under-five mortality prediction, Random Forest and Artificial Neural Network provides a balanced and effective strategy. Their consistent high performance, complementary strengths, and adaptability to complex patterns make them ideal candidates for practical deployment in healthcare settings. More research and validation are encouraged to improve the models’ interpretability and efficacy in a variety of real-world scenarios.

The findings of this study suggest that under-five mortality is a complex issue that is influenced by a range of social, economic, and demographic factors. The study found wealth index, maternal education, antenatal visits, place of delivery, employment status of the woman, children ever born, region, skilled assistance during delivery, frequency of watching television, and birth size to be the leading factors of under-five mortality. The study’s findings have important implications for public health policies and programs aimed at reducing under-five mortality. The findings suggest that targeted interventions are needed to address the specific needs and circumstances of different populations.

The future direction of our research would be to investigate advanced techniques for model optimization, interpretability, and ensemble learning, which can further refine the accuracy of under-five mortality predictions. We will also explore longitudinal analyses, which could track temporal trends and changes in under-five mortality rates over time, offering valuable insights into the evolving landscape of child health. Additionally, further research is needed that will assess the transferability of the predictive models to similar contexts and conduct external validation using diverse datasets, which would contribute to the generalizability of the findings.

## Data Availability

The dataset supporting the conclusions of this article is available in the IDHS repository. https://www.idhsdata.org/idhs-action/menu.

## Abbreviations

AUROC: Area Under the Receiver Operating Characteristics
ANN: Artificial Neural Network
FN: False Negative
FP: False Positive
FPR: False Positive Rate
k-NN: k-Nearest Neighbourhood
LR: Logistic Regression
ML: Machine learning
MCC: Matthews correlation coefficient
NB: Naïve Bayes
NDHS: Nigeria Demographic and Health Surveys
RF: Random Forest
SVM: Support Vector Machine
SMOTE: Synthetic Minority Oversampling Technique
TN: True Negative
TP: True positive
TPR: True Positive Rate
U5M: Under-five mortality
